# Network-based pharmacological study of the mechanism of Baitouweng decoction in the treatment of ulcerative colitis through the NF-κB pathway

**DOI:** 10.1097/MD.0000000000044167

**Published:** 2025-10-17

**Authors:** Shanze Bai, Baoxia Chen, Fang Li, Wanling Yao, Yanming Wei, Yongli Hua

**Affiliations:** a Institute of Traditional Chinese Veterinary Medicine, College of Veterinary Medicine, Gansu Agricultural University, Lanzhou, China.

**Keywords:** Baitouweng decoction, experimental verification, network pharmacology, ulcerative colitis

## Abstract

**Background:**

Baitouweng decoction (BD) is a classic formula for the treatment of ulcerative colitis (UC) with remarkable efficacy and low recurrence rate, although the therapeutic mechanism is still unclear. To screen out the active components and study the mechanisms of BD treating UC.

**Methods:**

A network pharmacology strategy was used to screen the active ingredients and pathways of action of BD for the treatment of UC, and the binding activity of the screened active ingredients to the core targets was examined by molecular docking with Autodock Vina software, a macrophage inflammation model was established, administration of BD and its screened active ingredients, determination of relevant inflammatory cytokine levels and expression of pathway-related proteins to verify the mechanism of action of BD in the treatment of UC.

**Results:**

Forty-five active components of BD, 186 relevant targets of UC, and 67 core intersecting genes of BD and UC were screened. Enrichment analysis revealed that the molecular mechanisms of BD treatment of UC are associated with IL-17, TNF-α, and Toll-like receptor signaling pathways, and that these signaling pathways all point to NF-κB-based inflammatory signaling pathways. Molecular docking suggests that the active components of BD, epiberberine, β-sitosterol, and anemoside B4 have a strong affinity for targets such as MPO, NOS3, and PPARG. MPO, NOS3, and PPARG can affect cytokine secretion and protein expression directly or indirectly through the NF-κB signaling pathway. Cell Counting Kit-8 results showed that BD and the active ingredient, in a range of nontoxic concentrations, increased cell survival in a model of LPS-induced cellular inflammation. BD and active ingredients could decrease LPS-induced inflammation and the levels of TNF-α, PGE2, IL-17, iNOS, IL-1β, COX-2, and NO. On the other hand, BD and the active ingredient promoted the expression and inhibited the phosphorylation of p65 and IκBα in the NF-κB signaling pathway in LPS-induced inflammation.

**Conclusions:**

β-sitosterol, stigmasterol, quercetin, isorhamnetin, berberine, and anemoside B4 may be the material basis for BD treatment of UC, which exerts anti-inflammatory effects by regulating the expression of inflammatory factors and modulating the NF-κB signaling pathway.

## 1. Introduction

Baitouweng decoction (BD is from “Shang Han Lun”) and consists of 4 herbs, *Pulsatilla chinensis* (Bunge) Regel (Bai Tou Weng), *Phellodendron chinense* C.K. Schneid. (Huang Bai), *Coptis chinensis* Franch. [Ranunculaceae] (Huang Lian), and *Fraxinus chinensis* Roxb. [Oleaceae] (Qin Pi). It has the effect of clearing heat and detoxifying toxins, cooling the blood to stop dysentery, commonly used in the treatment of all kinds of diarrhea, including bacillary dysentery and amebic dysentery.^[1]^ BD has antiviral and antibacterial, anti-inflammatory, anti-ulcer, anti-tumor, and anti-diarrheal effects.^[2–6]^

The main clinical manifestations of ulcerative colitis (UC) are diarrhea, purulent and bloody stools, urgency and heaviness, etc. It is characterized by a long course of disease, difficult to heal and easy to recur, which starts in the rectum and spreads to the colon and ileum with a variety of concomitant diseases (such as peritonitis), and is a chronic nonspecific intestinal inflammation.^[7,8]^A large number of modern experiment and clinical studies have shown that the efficacy of BD in treating UC is remarkable,^[9]^ but the molecular mechanism of its treatment of UC is still unclear.

Network pharmacology is the integrated use of biological and network information to explore the mechanism of drug treatment of diseases from the interactions of ingredients, targets, and diseases,^[10]^ for example, exploring the potential mechanism of HuanglianJiedu decoction against sepsis based on network pharmacology an exploration on mechanism of SimiaoYongan decoction in angiogenesis based on network pharmacology treatment,^[11]^ these research processes coincide with the pharmacological research process of Chinese medicine compound prescriptions. Molecular docking is a research method to predict the optimal binding mode of ligands and receptors, such as the use of network pharmacology and molecular docking to analysis on the anti-tumor effects of Andrographolide,^[12]^ such as using network pharmacology and molecular docking to explore the mechanism of the effect of LiuweiDihuang Pill on diabetic nephropathy,^[13]^ for the preliminary validation of network pharmacology results.

Therefore, this study utilized a network pharmacological approach and experimental validation to reveal the mechanism of action of BD in the treatment of UC, with a view to providing a theoretical basis for the treatment of UC with BD.

## 2. Methodology

### 2.1. Cells and medicinal materials

Mouse leukemia cells of monocyte macrophage (RAW264.7) was purchased from Procell Life Science & Technology Co., Ltd. *P chinensis, P chinense, C chinensis*, and *F chinensi*s were purchased from Lanzhou Yellow River Medicinal Material Market and identified by Professor Wei Yanming (College of Veterinary Medicine, Gansu Agricultural University, Lanzhou, China).

### 2.2. Main drugs and reagents

RAW264.7 cell-specific medium was purchased from Procell Life Science & Technology Co., Ltd. DMEM medium, fetal bovine serum was purchased from BI, Israel, 100X penicillin-streptomycin, Cell Counting Kit-8, dimethyl sulfoxide (DMSO), and bovine serum albumin (BSA) were purchased from Beijing Solarbao Technology Co. Stigmasterol (Yz101520,98%), β-sitosterol (Yz082523, 98%), quercetin (Yz011823, 99%), berberine (Yz110920, 99%), isorhamnetin (Yz090422, 98%), and anemoside B4 (Yz072922, 99%) were purchased from Nanjing Plant Origin Biotechnology Co., NO (A012-1-2), COX-2 (H200), and iNOS (H372-1) were purchased from Nanjing Jiancheng Bioengineering Institute Co., IL-17 (EMC008.96), IL-1β (EMC001b.96), and TNF-α (EMC102a.96) were purchased from Neobioscience Technology Co. Ltd. p65, IκBα antibodies, sheep anti-rabbit secondary antibody, and antibody dilutions were purchased from Beijing Bioss Biotechnology Co. p-p65 and p-IκBα antibodies were purchased from CST.

### 2.3. Instrument

Full-wavelength Enzyme Labeler (Beijing Putian Xinqiao Technology Co., Ltd.), inverted microscope (OLYMPUS, Japan), rotary evaporator (Shanghai Yarong Biochemical Instrument Factory), vacuum freeze dryer (Shanghai Heng Science Instruments Co., Ltd.), high-speed centrifuge (Shanghai Hetian Science Instruments Co., Ltd.), SDS-PAGE multifunctional electrophoresis instrument, vortex mixer (Shanghai Kanghua Biochemical Instrument Manufacturing Factory).

### 2.4. Network pharmacology

#### 2.4.1. Composition and target collection

PubChem and TCMSP databases were searched for the chemical constituents of BD, *P chinensis, P chinense, C chinensis*, and *F chinensis* and the screening conditions were oral bioavailability value ≥ 30% and drug-like properties value ≥ 0.18,^[6,7]^ the active ingredients of BD have been reported in the literature as supplements. The active ingredients of BD were obtained by these methods. The target genes of “UC” were searched in Pharmacogenetics and Pharmacogenomics Knowledge Base (PharmGKB database, https://www.pharmgkb.org/), DrugBank database (https://go.drugbank.com/drugs/), Online Mendelian Inheritance in Man (OMIM database, http://www.omim.org). The Uniprot protein database was used to convert human gene standard names with “*Homo sapiens* (Human)” as the species of the protein. The PDB protein structure database was used to search for BD component targets and UC disease targets.

#### 2.4.2. Construction of target network and UC target network of active ingredients of BD

Cytoscape software (3.9.0, The Cytoscape Consortium, LA Jolla) constructed interaction network diagrams for the screened BD active ingredient–target relationships, UC target relationships, respectively, and analyzed the core targets in the network by topological parameters, using the median values of degree, closeness centrality, and betweenness centrality as thresholds.

#### 2.4.3. Construction of protein interaction (PPI) networks and analysis of core targets

Protein interaction network analysis was performed by STRING11.0 database to obtain BD active ingredient action target protein interaction network (PPI) and UC disease target protein interaction network (PPI), and the TSV file was imported into Cytoscape software (3.9.0) to obtain the interaction network of the potential targets of BD for UC treatment, with a degree greater than the median node as the core intersection genes were obtained by the screening condition.

#### 2.4.4. Enrichment analysis

GO functional annotation and KEGG pathway analysis were performed for core intersection genes, and enriched genes were screened according to (*P* < .05),^[14]^ *q* < 0.05 (*q* is corrected *P*-value) using Rstudio software (RStudio, PBC, Boston) and R package (R Foundation for Statistical Computing, Vienna, Austria).

#### 2.4.5. Molecular docking verification

AutoDock Vina performed receptor ligand docking simulations on BD active components and core crossover genes. The strength of ligand–receptor binding was determined by Docking Score values, with free binding energy less than −16.736 kJ/mol indicating some binding activity, less than −20.920 kJ/mol indicating good binding activity, and less than −29.288 kJ/mol indicating strong binding activity.^[15–17]^ Molecular docking steric interaction maps were obtained using PyMOL software (Schrödinger, LLC, New York).

### 2.5. Preparation of BD aqueous extracts and BD alcoholic extracts

The extracts were made from *Pulsatilliae Radix, Phellodendri Chinrnsis Cortex, Coptidis Rhizoma*, and *Fraxini Cortex*, crushed and sifted through a 40 meshes, mixed in proportion of 4:3:2:4, 10 times (v/w) distilled water to soak the drugs for 1 hour, heated and refluxed for 1 hour, filtered to extract the supernatant, continued to heat and reflux the extracts 2 times (with 8, 8 times (v/w) water addition), decocted for 1 hour each time, combined the filtrates and concentrated under reduced pressure. Placed in a vacuum freeze dryer and freeze dry to obtain the final BD aqueous extract infusion (BDW).

The extracts were extracted from *Pulsatilliae Radix, Phellodendri Chinrnsis Cortex, Coptidis Rhizoma*, and *Fraxini Cortex*, crushed and sifted through 40 meshes, mixed in proportion of 4:3:2:4, soaked in 8 times (v/w) of 70% ethanol for 1 hour, decocted and refluxed for 1 hour. The supernatant was extracted by filtration and continued to be heated and refluxed for 2 times (adding 8 and 6 times (v/w) the amount of 70% ethanol), each time for 0.5 hours. The filtrates were combined and concentrated under reduced pressure. It was placed in a vacuum freeze dryer and freeze-dried to finally obtain BD alcohol extract infusion (BDC).

### 2.6. Cellular experimental

#### 2.6.1. Cell culture

RAW246.7 macrophages were cultured in 37°C, 100% humidity, 5% CO_2_ incubator. When the cells were spread 80% to 90%, the cells were blown off with a pipette gun to pass the culture, and the cells that could not be blown off were discarded.

#### 2.6.2. Cytotoxicity test

RAW246.7 macrophages were inoculated into 96-well plates at a density of 1 × 10^5^ cells/mL and placed in a 37°C, 100% humidity, 5% CO_2_ cell culture incubator for 24 hours. The experiments were grouped into different concentrations of DMSO (10%, 9%, 8%, 7%, 6%, 5%, 4%, 3%, 2%, 1%, 0.8%, 0.6%), chloroform (2%, 1%, 0.8%, 0.4%, 0.2%, 0.1%, 0.05%), 70% methanol (3%, 2%, 1%, 0.8%), BDW, BDC, quercetin, isorhamnetin (866, 433, 216, 108, 54, 27, 13 µg/mL), anemoside B4, berberine, β-sitosterol, stigmasterol (500, 250, 125, 62.5, 31.25 µg/mL), normal group (no cells, drug), and medication groups (no drug), after acting on the cells for 6 hours, Cell Counting Kit-8 10 µL was added to each well and the OD value was detected at 450 nm by enzyme marker after half an hour.

#### 2.6.3. ELISA assay for inflammatory factor levels

RAW246.7 macrophages were inoculated into 96-well plates at a density of 1 × 10^5^ cells/mL and placed in a 37°C, 100% humidity, 5% CO_2_ cell culture incubator for 24 hours. The wells were randomly divided into 3 groups, the normal group was treated with DMEM for 24 hours and then DMEM was replaced and continued for 4 hours, the control groups was treated with DMEM solution containing 1 µg/mL LPS for 24 hours, then DMEM was added to treat for 4 hours, the model groups were treated with DMEM solution containing 1 µg/mL LPS for 24 hours to construct LPS-induced cellular inflammation model, followed by the addition of drugs, with BDW high (433 µg/mL), medium (216 µg/mL), and low (108 µg/mL) concentrations in the experimental group, and BDC high (384 µg/mL), medium (171 µg/mL), low (76 µg/mL) concentrations (DMSO <2%), 250 µg/mL of β-sitosterol, stigmasterol solution (chloroform <1%), 384 µg/mL of isorhamnetin and quercetin solution, 62.5 µg/mL of anemoside B4 solution (DMSO <2%), and 500 µg/mL of berberine solution (70% methanol <3%). Six replicate wells were provided for each concentration, and the supernatant was collected in a sterile centrifuge tube after 4 hours of action, centrifuged at 2500 rpm for 15 minutes, and the supernatant was aspirated in a sterile centrifuge tube and stored at −20℃. The content of TNF-α, IL-17, IL-1β, TNF-α, PGE2, NO, iNOS, and COX-2 in each group was detected by ELISA kit.

#### 2.6.4. Protein expression of p65, p-p65, IκBα, p-IκBα in cells by protein immunoblotting

RAW246.7 macrophages were inoculated into 6-well plates at a density of 2 × 10^6^ cells/mL, and the cell culture and modeling procedure was the same as in section 2.6.3. The model groups were those containing BDW, BDC high and medium concentrations, β-sitosterol, stigmasterol, isorhamnetin, quercetin, anemoside B4, and berberine at the same concentration as in section 2.6.3. And 6 replicate wells were provided for each concentration. After continued incubation in the incubator for 4 hours, cellular protein extraction was performed. After protein denaturation treatment, electrophoresis, membrane transfer, closure and incubation with antibodies P65 (1:1000), p-P65 (1:1000), IκBα (1:1000), p-IκBα (1:1000), and β-actin (1:5000) were performed overnight. After incubation with horseradish peroxidase-labeled secondary antibodies, ECL chemiluminescence was developed and the target protein expression levels were calculated by Image J software (GraphPad Software, Inc., Boston).

### 2.7. Statistical analysis

All statistical analyses and graphing were performed by SPSS 26.0 (IBM Corp., Armonk) and Graphpad prism software (GraphPad Software, Inc., Boston). Measures were expressed as mean plus or minus standard deviation ($\bar{X}$ ± SD), and when normality and variance chi-squared are met, 1-way ANOVA was used for within-group comparisons and uncorrected LSD test for between-group comparisons, *P* < .05, that is, significant differences between groups, and *P* < .01, that is, extremely significant differences, were statistically significant.

## 3. Results

### 3.1. Network pharmacology analysis

#### 3.1.1. Screening of core targets and active ingredient–target network of BD

**Figure 1.**
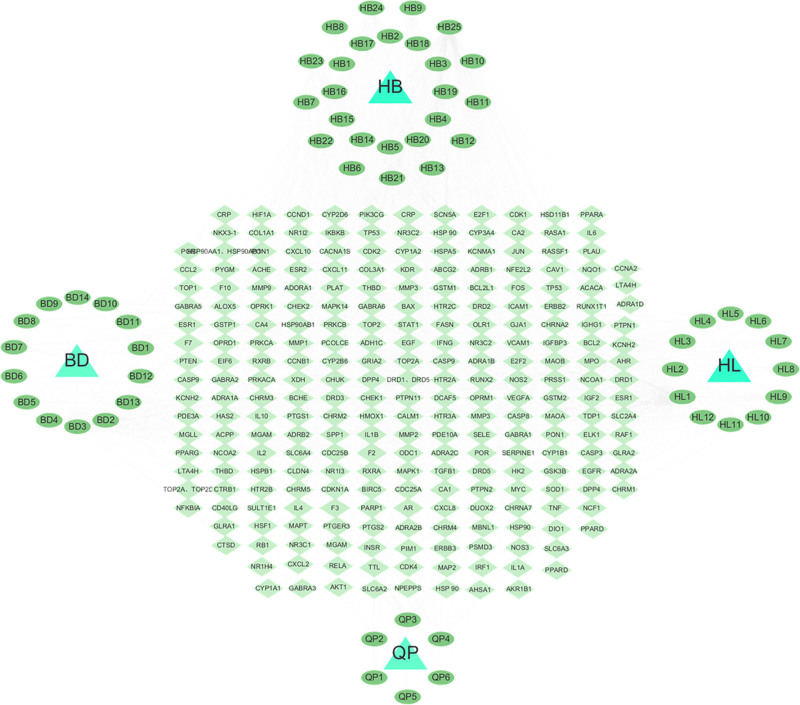
Active ingredientis–target–disease network of Baitouweng decoction. Triangle: *Pulsatilla chinensis* (BD), *Fraxinus chinensis* (QP), *Phellodendron chinense* (HB), *Coptis chinensis* (HL); Ellipse (outer): active components of Baitouweng decoction; Ellipse (inside): the target of BD acting on UC. BD = Baitouweng decoction, UC = ulcerative colitis.

Forty-five active ingredients of BD were screened, among which 12 active ingredients were attributed to *P chinensis*, 6 to *F chinensis*, 12 to *C chinensis*, and 25 to *P chinense*. The Cytoscape (3.9.0) software was imported to construct the active ingredient–target network of BD (Fig. 1). The active ingredients of BD were screened by closeness centrality, betweenness centrality, and degree values (Table 1). Quercetin, β-sitosterol, isorhamnetin, berberrubine, stigmasterol, anemoside B4 ranked high in terms of the number of potential targets. A higher value of degree means that the node has more connected nodes in the network.^[18]^

**Table 1:** Active ingredients of Radix Pulsatilla decoction.

Herbs	ID	Compound	CAS	Degree	OB	DL	BC	CC
Pulsatilliae Radix	BD2	Isorhamnetin	480-19-3	37	49.6	0.31	0.06589511	0.38124239
	BD3	Beta-sitosterol	83-46-5	38	36.91	0.75	0.04543589	0.38594328
	BD4	Stigmasterol	83-48-7	32	43.83	0.76	0.04579716	0.37847642
	BD10	Anemoside B4	–	18	6.79	0.01	0.01864303	0.35853379
Coptidis Rhizoma	HL1	Quercetin	-10-1	151	46.43	0.28	0.33295529	0.51907131
	HL6	Worenine	-29-0	8	45.83	0.87	0.00158583	0.33475936
	HB5	Palmatine	3486-67-7	29	64.6	0.65	0.01793468	0.36018412
Phellodendri Cortex	HB19	Chelerythrine	–	37	34.18	0.78	0.03741868	0.36780259
	HB23	Berberrubine	-69-1	32	35.74	0.73	0.03892491	0.3704142
	HB24	Campesterol	474-62-4	38	37.58	0.71	0.03985107	0.38217338
	HB25	Thalifendine	-71-1	151	44.41	0.73	0.34173623	0.52253756
Fraxini Cortex	QP1	beta-sitosterol	83-46-5	38	36.91	0.75	0.00476894	0.33764833
	QP3	8-(beta-D-Glucopyranosyloxy)-7-hydroxy-6-methoxy-2H-1-benzopyran-2-one	524-30-1	29	36.76	0.42	0.03128936	0.371293

BC = betweenness centrality, CC = closeness centrality, DL = drug-like properties, OB = oral bioavailability.

#### 3.1.2. UC target network construction

**Figure 2.**
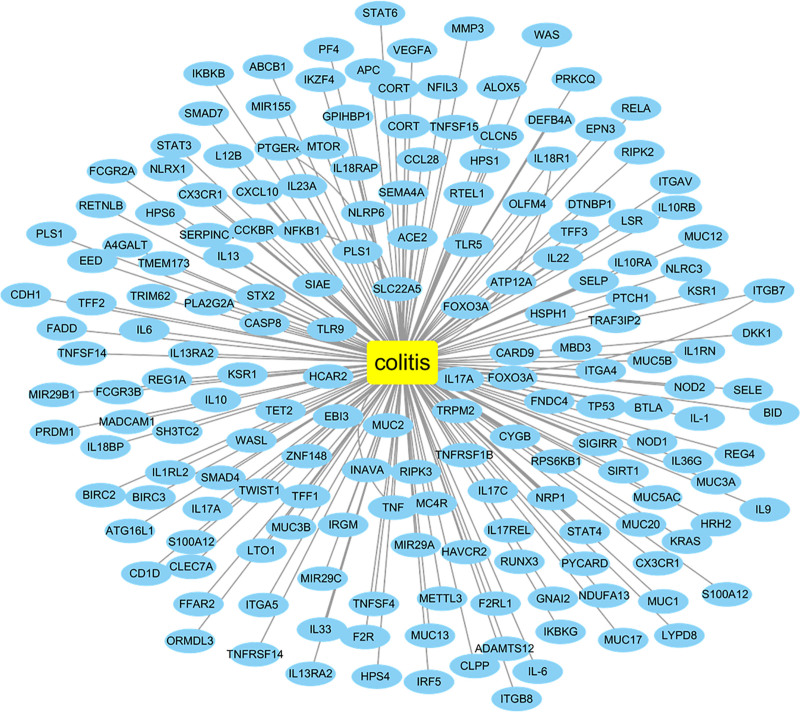
Ulcerative colitis–target network the rectangle represents UC itself and the ellipse represents UC gene targets. UC = ulcerative colitis.

UC targets were selected from DrugBank, OMIM, and PharmGKB databases, and a total of 186 UC targets were obtained after removing duplicate values (Fig. 2).

#### 3.1.3. The targets for the treatment of UC with BD

**Figure 3.**
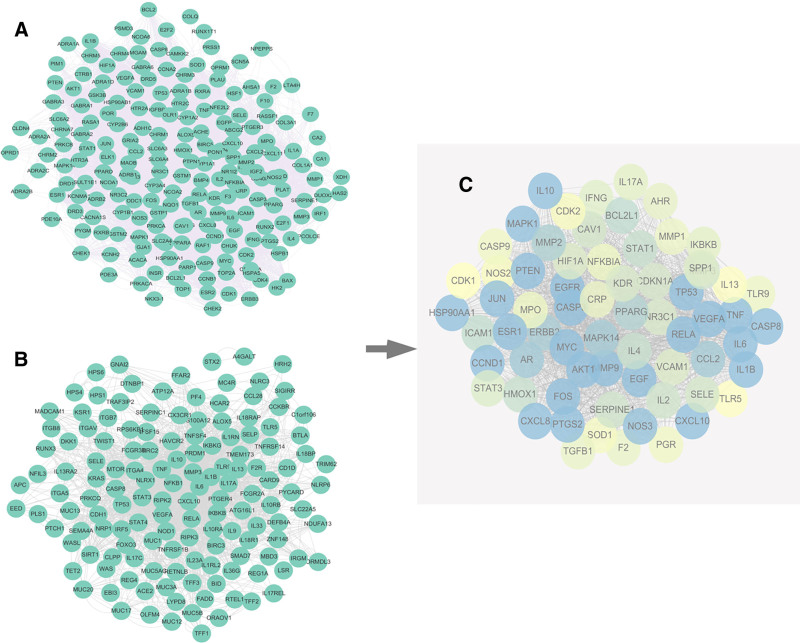
Potential interaction target network of BD therapy UC. (A) Effect of BD on PPI network of ulcerative colitis; (B) PPI network of gene targets for ulcerative colitis; (C) network of potential targets of BD in the treatment of ulcerative colitis (*Note*: the color shift from yellow to blue represents the degree of protein interconnection, the bluer the color, the higher the degree of protein interconnection.). BD = Baitouweng decoction, UC = ulcerative colitis.

The BD active ingredient–target protein interaction network and UC gene target protein interaction network were obtained from STRING11.0 database and Cytoscape (3.9.0) software (Fig. 3A and B). The potential interaction target network of BD for treating UC and its core intersection target network of BD and UC were obtained using Cytoscape (3.9.0) and its plug-in (Fig. 3C), degree with there are 67 targets of BD active ingredients for the treatment of UC screened at a threshold greater than the median 45, among which targets greater than twice the degree are in a central position in BD for UC and may play important therapeutic roles, including IL-6, TNF, TP53, VEGFA, AKT1, IL-10, IL-1β, RELA, CASP3, MAPK1, JUN, EGFR, MYC, EGF FOS, ESR1, and PTGS2.

#### 3.1.4. Enrichment analysis

**Figure 4.**
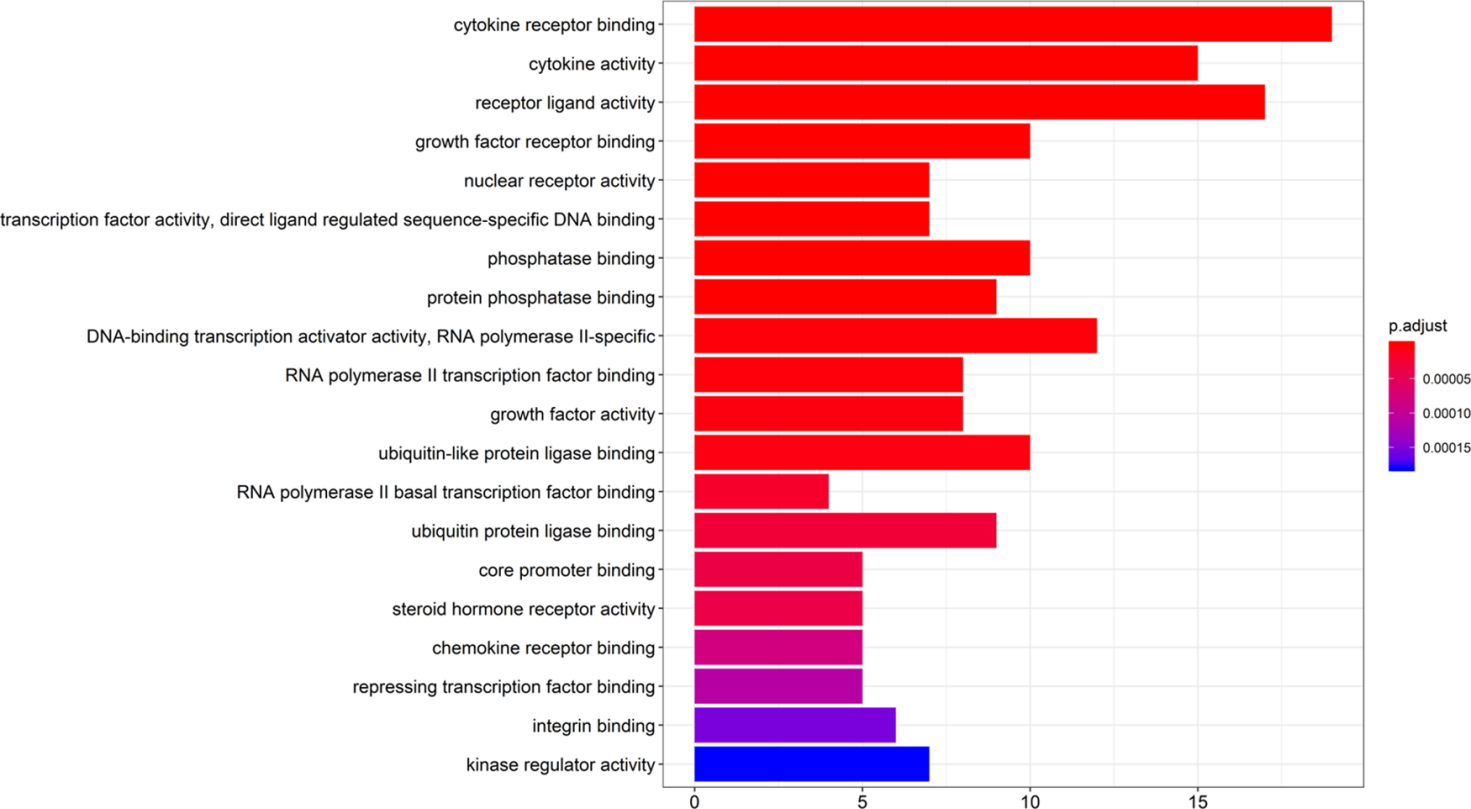
Results of GO enrichment analysis. *Note*: the color represents the *P*-value, and the darker the color, the smaller the *P*-value, the more significant the pathway.

For the target biological function enrichment analysis, a threshold value of less than *P* < .05 was set, 114 biological processes were obtained, and the top 20 GO entries were plotted from the smallest to the largest *P* values (Fig. 4). The top enriched genes include cytokine receptor binding, cytokine activity, receptor ligand activity, phosphatase binding, growth factor activity, ubiquitin-like protein ligase binding, core promoter binding, chemokine receptor binding, etc. Among them, cytokine receptor binding, cytokine activity, and receptor ligand activity genes were enriched in numbers >15, and these biological processes may play an important role in the treatment of UC by BD.

**Figure 5.**
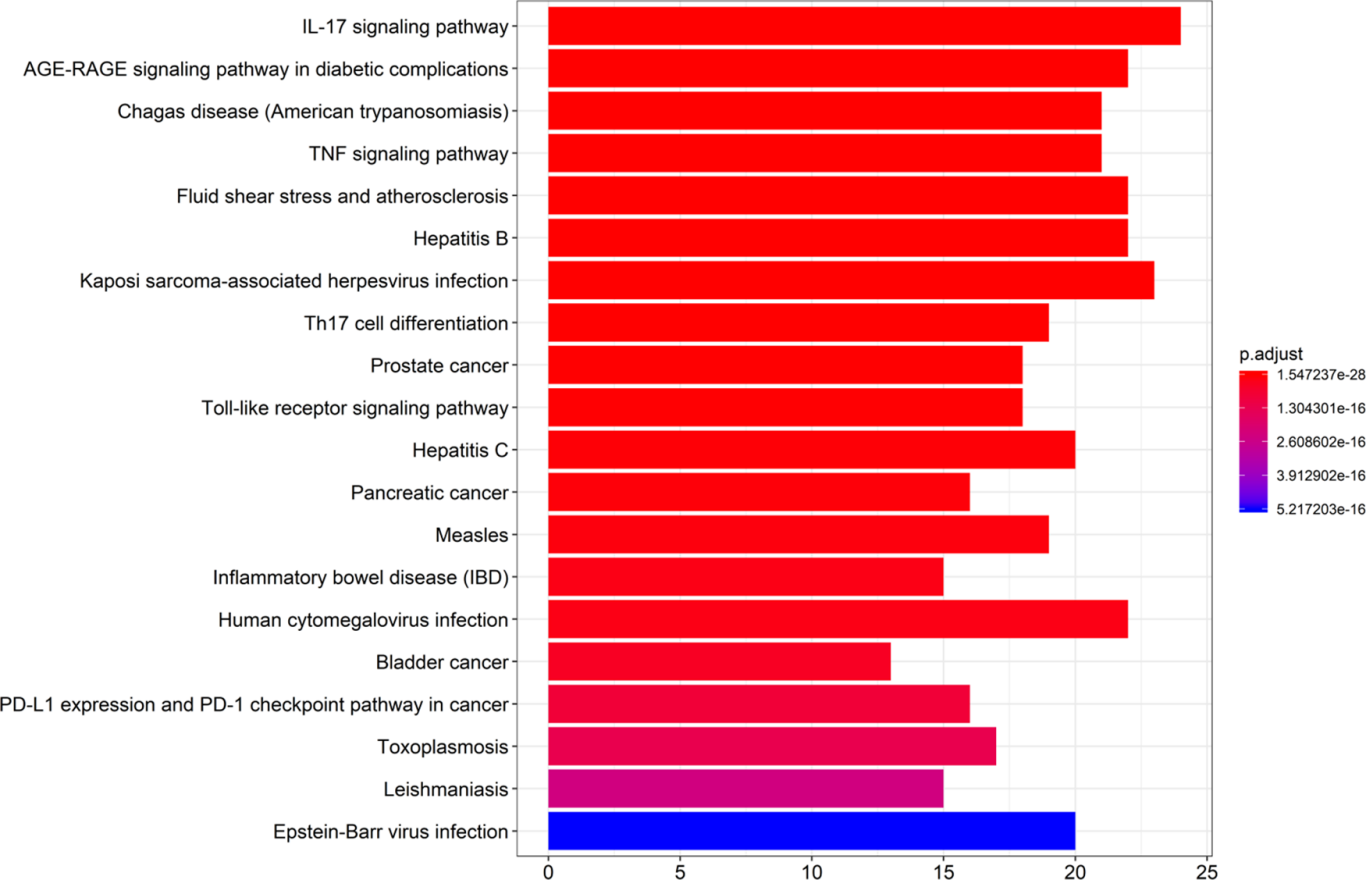
Results of KEGG enrichment analysis. *Note*: the color represents the *P*-value, and the darker the color, the smaller the *P*-value, the more significant the pathway.

Pathway enrichment analysis was performed for the core targets, and a threshold value of less than *P* < .05 was set to obtain 134 signaling pathways, and the top 20 KEGG entries were ranked from smallest to largest *P* values for the graphs (Fig. 5), in which the signaling pathways associated with UC were mainly IL-17 signaling pathway, TNF signaling pathway, Toll-like receptor signaling pathway, IBD, Th17 cell differentiation, and tumor-related pathways. Among them, IL-17 signaling pathway, TNF signaling pathway gene enrichment number is >20, and both of these pathways are associated with the treatment of inflammation. These signaling pathways may play an important role in the treatment of UC in BD. And among them, IL-17 signaling pathway, TNF signaling pathway and Toll-like receptor signaling pathway are all attributed to NF-κB-based inflammatory signaling pathway, indicating that NF-κB signaling pathway plays a significant role in the treatment of UC by BD.

#### 3.1.5. Molecular docking validation results

**Figure 6.**
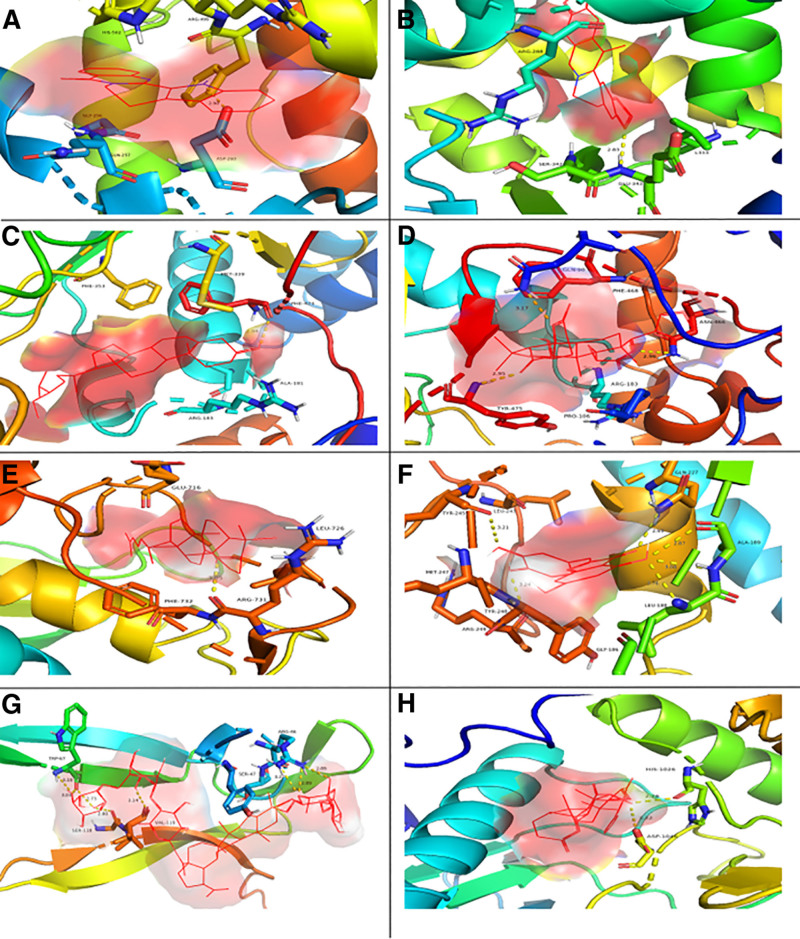
Molecular docking results. *Note*: active compounds are represented by ball-and-stick model, the secondary structure of the protein was represented by ribbon. The yellow dotted liners represent hydrogen bond.

The results of the screening of 45 molecular docking of BD active ingredients for the treatment of UC showed that the binding free energy ranged from −12.552 kJ/mol to −49.789 kJ/mol, and the number of binding BD active ingredients exceeded 10 and the binding free energy was less than −37.656 kJ/mol as the threshold value to obtain 9 core intersection genes, in the order of cell cycle protein CDK1, EGFR, KDR, MAPK1, MMP3, MMP9, MPO, NOS3, PPARG, BD active components, and these genes have strong binding activity. Among them, MPO and rutaecarpine, PPARG and worenine, NOS3 and β-sitosterol, epiberberin, MMP3 and LAN, MMP9 and aureusidin, IL-17A and anemoside B4, KDR, and epiberberine in BD the possible therapeutic role in the treatment of UC was mapped for their molecular docking results (Fig. 6).

### 3.2. Experiment verification

#### 3.2.1. BD and active ingredients, DMSO, chloroform, 70% methanol cytotoxicity assay

**Figure 7.**
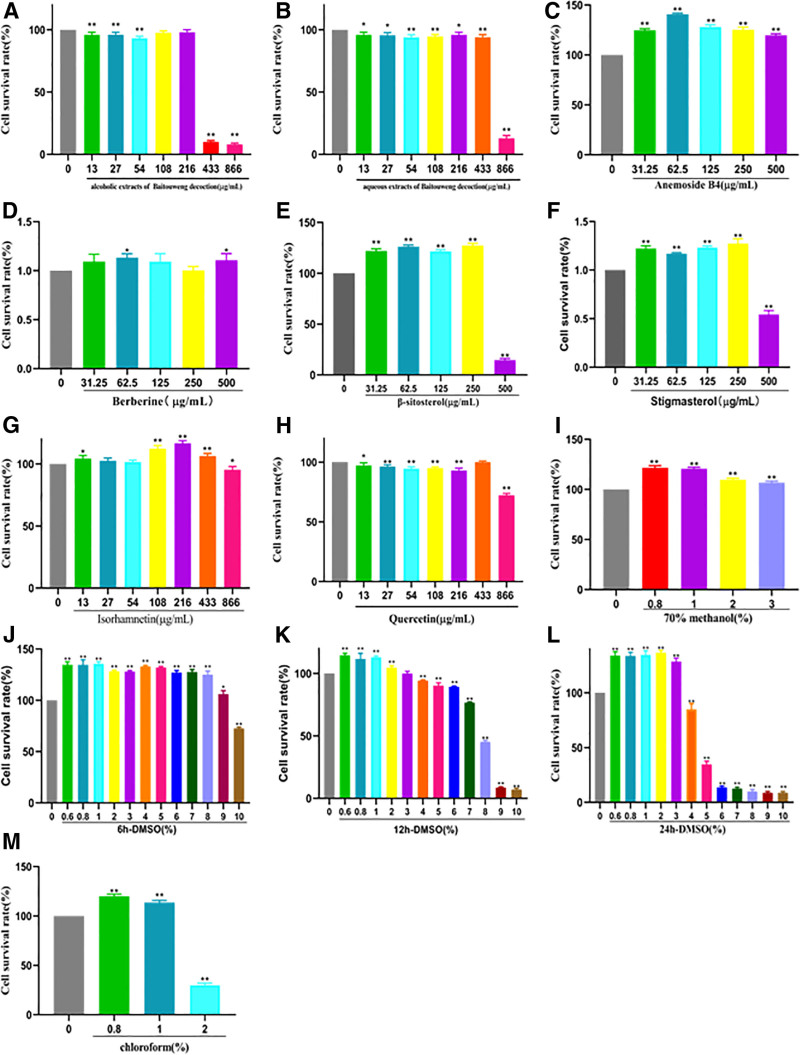
In vitro analysis of the cytotoxic effects of BD and its active ingredient, the reagent that dissolves the drug, BD and its active ingredient, the reagent that dissolves the drug. Comparison with blank group **P < .01, *P < .05. A, B, C, D, E, F, G, H, I, J, K, L, M is the toxic effect of A (BD alcohol extract), B (BD water extract), C (anemoside B4), D (berberine), E (β-sitosterol), F (stigmasterol), G (isorhamnetin), H (quercetin), I (70% methanol), J (6 hour DMSO), K (12 hour DMSO), L (24 hour DMSO), and M (chloroform) on RAW264.7 cells.

As shown in Figure 7, the toxicity of BDW, BDC, and BD active ingredient, DMSO, chloroform, and 70% HPLC methanol on macrophages increases with the increase of action concentration and time. No significant cytotoxicity at BDW concentration below 433 µg/mL, no significant cytotoxicity at BDC concentration below 216 µg/mL, 866 µg/mL were the most cytotoxic, and 433, 216, and 108 µg/mL were selected as the high, medium, and low concentrations of BDW and 216, 108, and 54 µg/mL were selected as the high, medium, and low concentrations of BDC, which were not significantly toxic to cells and had the highest cell viability. The concentrations of anemoside B4 and berberine below 500 µg/mL, β-sitosterol and stigmasterol below 250 µg/mL, isorhamnetin and quercetin below 433 µg/mL were not significantly toxic to cells, and combined with the cell viability results, 62.5, 500, 216, 433, 250, and 250 µg/mL as the safe action concentrations of anemoside B4, berberine, isorhamnetin, quercetin, β-sitosterol, and stigmasterol. DMEM solution containing DMSO had no significant cytotoxicity at concentrations lower than 9%, 6%, and 2% at 6, 12, and 24 hours. The concentrations of 2% and below were selected as the safe action concentration range of DMSO when combined with cell viability DMSO action cells for 24 hours. DMEM solution containing chloroform, DMEM solution containing 70% methanol are not significantly cytotoxic when it is <1% and 3% or less, respectively, so less than or equal to 1% is selected as the safe concentration range of chloroform and less than or equal to 3% as the safe concentration range of 70% methanol.

#### 3.2.2. Effects of BD and its active ingredients on cytokines

**Figure 8.**
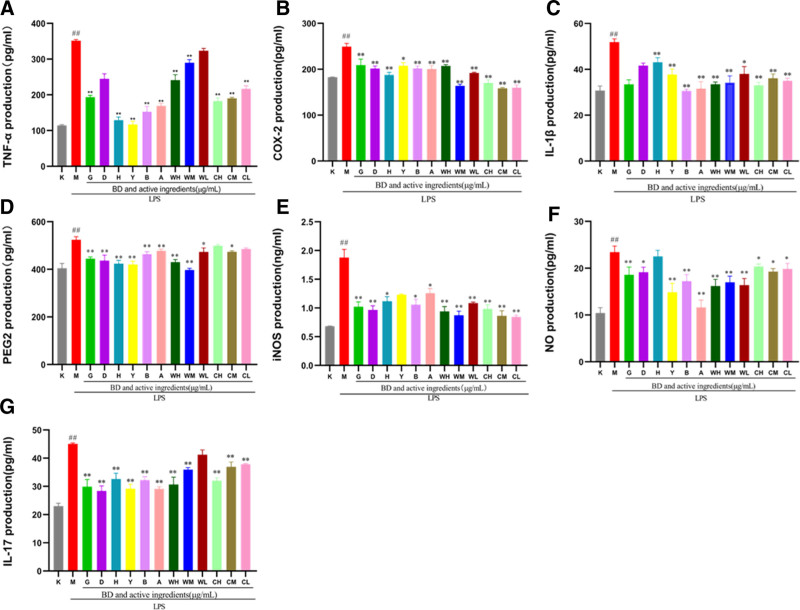
In vitro analysis of the effect of BD and its active components on cytokines in a model of LPS-induced cellular inflammation. Compared with the K group: ## *P* < .01; compared with M group: **P < .01, * *P* < .05 (logo as shown in this figure) (n = 3). A, B, C, D, E, F, and G are the contents of TNF-α, COX-2, IL-1β, PEG2, iNOS, NO, and IL-17 in the supernatant of RAW264.7 cells. K (the normal group), M (model group), G (β-sitosterol), D (stigmasterol), H (quercetin), Y (isorhamnetin), B (berberine), A (anemoside B4), WH (BD water extract high concentration), WM (BD water extract medium concentration), WL (BD water extract low concentration), CH (BD alcohol extract high concentration), CM (BD alcohol extract medium concentration), and CL (BD alcohol extract low concentration).

Compared with the normal group, the TNF-α, IL-1β, PGE2, IL-17, iNOS, COX-2, and NO contents in the model group were significantly increased (Fig. 8). The difference between cytokine secretion levels in the model group and the normal group was significant. β-Sitosterol, stigmasterol, quercetin, isorhamnetin, berberine, anemoside B4, BDW, and BDC at high and medium concentrations reduced the secretion of each cytokine, among which, β-sitosterol and stigmasterol reduces iNOS, IL-17 expression is the best, isorhamnetin reduces NO, COX-2, IL-17, PGE2, TNF-α expression effect is the best, berberine reduces IL-1β, TNF-α expression effect is the best, anemoside B4 reduces TNF-α, IL-1β, NO, IL-17 expression effect is the best, BD reduces IL-1β, iNOS, COX-2, NO, PGE2 expression is the best, and BDC reduces TNF-α, IL-17, IL-1β, INOS, COX-2 expression effect is better than BDW (*P* < .01).

#### 3.2.3. Effect of BD and active ingredients on the expression of p65 protein, p-p65 protein, IκBα protein, and p-IκBα protein

**Figure 9.**
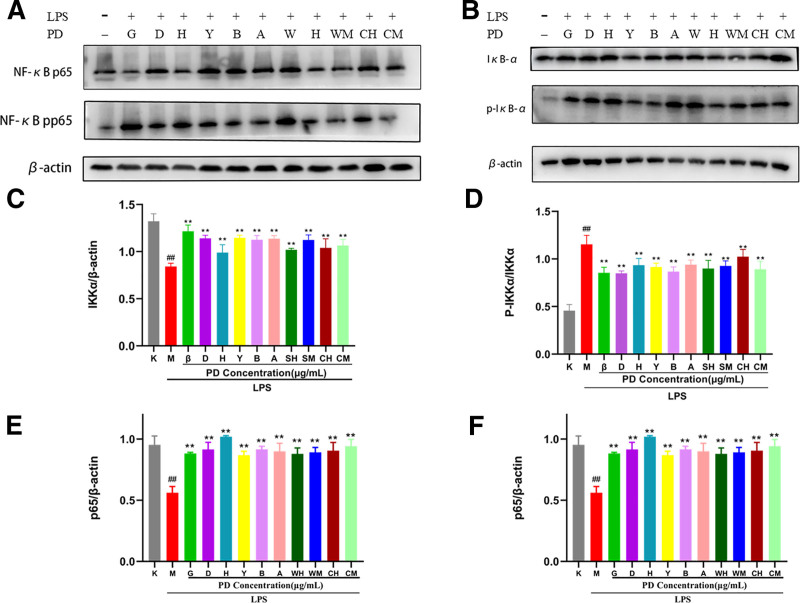
Effect of BD and its active ingredients on the expression of p65, IκBα, and its phosphorylated protein. Compared with the K group: ## *P* < .01; compared with M group: **P < .01, * *P* < .05 (logo as shown in this figure). K (the normal group), M (model group), G (β-sitosterol), D (stigmasterol), H (quercetin), Y (isorhamnetin), B (berberine), A (anemoside B4), WH (BD water extract high concentration), WM (BD water extract medium concentration), CH (BD alcohol extract high concentration), and CM (BD alcohol extract medium concentration).

LPS treatment of RAW264: 7 cells for 24 hours, the expression of p65 and IκBα total protein was significantly decreased in the model group compared with the normal group (*P* < .01), and the expression of p-p65 and p-IκBα proteins increased (*P* < .01); after continued treatment of cells with BDW, BDC, and active ingredients for 4 hours, all groups were able to upregulate the expression of p65 and IκBα proteins compared with the model group (*P* < .01) and down-regulated the expression of p-p65 and p-IκBα proteins (*P* < .01) (Fig. 9). Among them, berberine and BDC medium concentration inhibited p-p65 expression better, quercetin and BDC medium concentration promoted p65 expression better. BDC promoted p65 expression and inhibited its phosphorylation better than BDW. Stigmasterol, β-sitosterol, berberine, BDW high concentration, and BDC medium concentration inhibited p-IκBα expression better. Better promotion of IκBα expression by of β-sitosterol, stigmasterol, isorhamnetin, anemoside B4, BDW medium concentrations. BDW promotes the expression of IκBα and inhibits its phosphorylation better than BDC.

## 4. Discussion

Through the study of network pharmacology, it was found that β-sitosterol, stigmasterol, quercetin, isorhamnetin, berberine, and anemoside B4 are the material basis of BD for the treatment of UC. Quercetin belongs to the group of flavonoids, which can inhibit the activation of NF-κB pathway to exert analgesic and anti-inflammatory effects, as well as improve diarrhoeal status.^[19,20]^ Quercetin exerts powerful anti-inflammatory effects by acting on the Nod-like receptor protein 3 inflammasome.^[21]^ Isorhamnetin, a direct derivative of quercetin, can upregulate the expression of the human PXR gene, it can inhibit the activity of MPO, block the release of pro-inflammatory mediators (TNF-α, IL-6, COX-2, iNOS, etc), act on the NF-κB signaling pathway to achieve anti-inflammatory and therapeutic effects in inflammatory bowel disease effects,^[22]^ isorhamnetin could suppress TNF-α-induced apoptosis and inflammation by blocking NF-κB and AP-1 signaling.^[23]^ β-sitosterol and stigmasterol are sterols that exhibit anti-inflammatory effects through diverse mechanisms. β-sitosterol enhances IL-10 anti-inflammatory activity, reduces chemokines and pro-inflammatory factors, inhibits NF-κB activation, and decreases NO synthesis and IL-6 activity while lowering TNF-α secretion. Stigmasterol suppresses the mRNA expression of IL-1, IL-6, MCP-1, and COX-2, contributing to its anti-inflammatory properties.^[24]^ Berberine belongs to the group of alkaloids, and it ameliorates DSS-induced colonic injury in UC mice,^[25]^ Berberine exerts its anti-inflammatory effects through multiple pathways, including inhibition of pro-inflammatory cytokines (e.g., IL-1β, IL-6, and TNF-α), modulation of NF-κB, MAPK, and STAT1 signaling, and interaction with cellular membranes to influence cellular activities.^[26]^ Anemoside B4 effectively ameliorate experimental UC mainly through regulating MLCK/pMLC2 pathway and ameliorates TNBS-induced colitis through S100A9/MAPK/NF-κB signaling pathway.^[27,28]^ The above analysis indicated that all the compounds screened in this study had anti-inflammatory activity, and verified that the results were reasonable, and these compounds were the active ingredients of BD.

Existing studies also have demonstrated that BD and β-sitosterol, stigmasterol, quercetin, isorhamnetin, berberine, and anemoside B4 affect the intestinal immune barrier in an indirect or direct manner by regulating the expression of genes in the signaling pathway and regulating the activity and release of corresponding cytokines or enzymes, affecting lymphocytes and thus regulating intestinal immune homeostasis, regulating the inflammatory response, and exerting anti-inflammatory, ameliorating intestinal mucosal damage and protecting the intestine,^[29,30]^ which is consistent with the results of network pharmacology and molecular docking analysis in this experiment. Therefore, it is speculated that the mechanism of action of BD in treating UC is through regulating the secretion and activity of cytokines and acting on inflammatory signaling pathways such as NF-κB to exert anti-inflammatory effects and improve diarrheal symptoms.

TNF-α regulates the expression of relevant inflammatory proteins in cells and induces neutrophils to act,^[15]^ activating cells to produce inflammatory factors and thus causing inflammation.^[16]^ TNF-α in turn enhances the activation of NF-κB, creating a feedback loop of inflammation.^[17]^ Isorhamnetin can inhibit the release of NO after LPS stimulation and thus relieve pain, affect the NF-κB pathway, inhibit the release or expression of TNF-α after LPS stimulation, and thus exert analgesic effects,^[31]^ quercetin can significantly regulate the serum level of TNF-α in rats with chronic prostatitis for anti-inflammatory purposes.^[32]^ The level of TNF-α responds to the severity of UC and decreases in serum after PD treatment.^[33]^ In this experiment, isorhamnetin and quercetin had the best effect on inhibiting TNF-α secretion in the LPS-induced macrophage inflammation model, suggesting that isorhamnetin and quercetin mainly inhibit TNF-α secretion, then inhibit activation of NF-κB pathway to achieve anti-inflammatory and analgesic effects. An acute immune response occurs and iNOS is activated to produce large amounts of NO. Clinical studies have shown that inhibition of the iNOS enzyme blocks the immune response in vivo, when this was applied to UC, it was found that NO secretion at the colonic mucosa was significantly elevated during the disease, as was iNOS.^[16]^ β-sitosterol can inhibit iNOS expression to alleviate endothelial inflammation and cellular damage caused by high glucose.^[34]^ Berberine inhibits the transcription and translation of iNOS to alleviate intestinal inflammation,^[35]^ and also inhibits NO secretion for anti-inflammatory purposes.^[36]^ Saponins can regulate the activation of NF-κB pathway to inhibit NO expression to anti-inflammatory.^[37]^ In this experiment, BDW and stigmasterol inhibited iNOS secretion best, and anemoside B4, isorhamnetin, and BDW inhibited NO secretion best, suggesting that anemoside B4, isorhamnetin, and BDW mainly inhibited NO secretion then exerted anti-inflammatory effects, on the one hand BDW and stigmasterol mainly inhibit the secretion of iNOS and promote the normal immune response, and inhibit NO secretion to alleviate inflammatory damage on the other hand. Cyclooxygenase plays an essential role in the synthesis of prostaglandins, and the synthesis of PGS is triggered by the massive expression of COX-2 after external stimuli, when acute inflammation occurs, PGS is the fastest way to initiate leukocyte aggregation and immune cell infiltration.^[38]^ The clinical activity of the UC condition shows a positive correlation with the amount of COX-2 secretion.^[16]^ Quercetin, a selective inhibitor of COX-2, regulates inflammation^[39]^ and inhibits the secretion of PGE2 to impede the inflammatory response.^[40]^ Isorhamnetin blocks NF-κB pathway to make COX-2 secretion decrease to treat enteritis.^[41]^ In this experiment, BDW, quercetin, and isorhamnetin inhibit PGE2 secretion best, and BDW, BDC, and quercetin inhibit COX-2 secretion best, suggesting that BDW, quercetin, and isorhamnetin mainly inhibit PGE2 secretion and thus prevent the continuous development of inflammatory response. BDW, BDC, and quercetin mainly inhibited COX-2 secretion, on the one hand, they directly to inhibit the continued deterioration of UC disease, on the other hand, they prevented PGS synthesis, regulated immune cell response and hindered inflammation. The expression of IL-1β is higher in UC patients than in normals and the disease progression and drug efficacy of UC are positively correlated with the expression level of IL-1β.^[17]^ Berberine down-regulated the expression of TNF-α and IL-1β in colonic tissues and effectively improved colitis lesions.^[42]^ Anemoside interfered with NALP3 pathway to downre-gulate IL-1β secretion as an anti-inflammatory agent.^[43]^ In this experiment, berberine, anemoside B4, BDW high and medium concentrations, and BDC inhibited IL-1β secretion the best, suggesting that berberine, anemoside B4, BDW high and medium concentrations, and BDC mainly inhibited IL-1β secretion, repaired intestinal pathological changes, and played anti-inflammatory effects. Sterols inhibit the secretion of NO and thus the release of IL-17,^[44]^ flavonoids regulate inflammation by inhibiting the production of inflammatory cytokines and binding to receptors,^[45]^ and saponins modulate MAPK activity to affect the secretion of IL-17 and thus regulate inflammation.^[46]^ IL-17 is pro-inflammatory in addition to other pro-inflammatory factors such as IL-1β and TNF-α, promotes repair of the epithelial barrier and intestinal epithelial crypts, prolonged and massive activation of IL-17 can cause immune dysregulation in the intestine.^[47]^ In this experiment, the best inhibitory effect of IL-17 secretion was observed by stigmasterol, isorhamnetin and anemoside B4, suggesting that stigmasterol, isorhamnetin, and anemoside B4 mainly inhibit IL-17 secretion, suppress the development of pro-inflammatory response, promote intestinal epithelial repair, and maintain intestinal immune homeostasis.

The studies have demonstrated that berberine down-regulates the transcription and translation of NF-κB p65, hinders its entry into the nucleus to turn on the expression of inflammatory genes,^[48,49]^ quercetin upregulates the protein level of NF-κB p65 in LPS-stimulated cells,^[50]^ saponins up-regulate the expression level of IκBα when used alone or in combination in mouse tests,^[51]^ berberine treatment of rat p-IκBα in liver tissues was significantly reduced after inflammation,^[52]^ sterols obtained by isolation and purification effectively increased cellular IκBα protein expression in cellular inflammation models and down-regulated p-p65 and p-IκBα expression levels and thus anti-inflammatory.^[53]^ In RAW264.7 macrophages stimulated by LPS, p65 protein translocated to the nucleus in large amounts, driving the expression of related genes, and IκBα phosphorylated then detached from the dimer, mediating and stimulating downstream signaling. In this experiment BD and its active components promoted the expression of p65 and IκBα and inhibited their phosphorylation, suggesting that on the one hand BD and its active components may promote the expression of p65 and IκBα, on the other hand it inhibits p65 phosphorylation to prevent it from driving the expression of related genes, inhibits IκBα phosphorylation to prevent it from mediating and stimulating downstream signaling after detaching from the dimer, thus exerting therapeutic effects to alleviate the symptoms of diarrhea and abdominal pain, ameliorating inflammatory damage in the intestinal tract and maintain intestinal immune homeostasis.

### 4.1. Limitations of the study

This study focused on using network pharmacology to screen the anti-inflammatory chemical components of BD, and establishing a cell inflammation model to verify the anti-inflammatory effect of BD and the selected active ingredients. However, animal experimental models in vivo need to be established to verify its anti-inflammatory effect, and the anti-inflammatory mechanism needs to be studied in more detail.

## 5. Conclusion

The mechanism of action of BD in the treatment of UC may be based on the substance of β-sitosterol, stigmasterol, quercetin, isorhamnetin, berberine, and anemoside B4, which act on the NF-κB signaling pathway to achieve anti-inflammatory effects and exert therapeutic effects by regulating the secretion of cytokines, modulating the expression of p65 and IκBα proteins, and inhibiting their phosphorylation. In this study, we applied the network pharmacology analysis strategy to elucidate the therapeutic effect of BD through multi-component, multi-target, and multi-pathway interactions for the treatment of UC, and verified the effect of aqueous and alcoholic extracts of BD and its active ingredients β-sitosterol, stigmasterol, quercetin, isorhamnetin, berberine, and anemoside B4 for the treatment of UC by cellular assay, which provides the basis and theory for the clinical application of BD for UC treatment.

## Author contributions

**Conceptualization:** Shanze Bai, Yongli Hua.

**Data curation:** Shanze Bai.

**Formal analysis:** Shanze Bai.

**Investigation:** Baoxia Chen, Fang Li.

**Methodology:** Shanze Bai, Baoxia Chen, Fang Li.

**Resources:** Shanze Bai.

**Software:** Shanze Bai.

**Validation:** Shanze Bai.

**Visualization:** Shanze Bai.

**Writing – review & editing:** Wanling Yao, Yanming Wei, Yongli Hua.

**Writing – original draft:** Yanming Wei, Yongli Hua.
